# Historical long-term cultivar×climate suitability data to inform viticultural adaptation to climate change

**DOI:** 10.1038/s41597-022-01367-6

**Published:** 2022-06-06

**Authors:** Huiqing Bai, Gregory A. Gambetta, Yongjian Wang, Junhua Kong, Qinqin Long, Peige Fan, Wei Duan, Zhenchang Liang, Zhanwu Dai

**Affiliations:** 1 Beijing Key Laboratory of Grape Science and Enology and Key Laboratory of Plant Resources, Institute of Botany, The Chinese Academy of Sciences, Beijing, 100093 China; 2 EGFV, Bordeaux-Sciences Agro, INRAe, Université de Bordeaux, ISVV, 210 Chemin de Leysotte 33882 Villenave d’Ornon, Bordeaux, France

**Keywords:** Phenology, Phenology

## Abstract

Grape quality is regulated by complex interactions between environments and cultivars. Growing suitable cultivars in a given region is essential for maintaining viticulture sustainability, particularly in the face of climate change. We created a database composed of three different subsets of data. The first subset was created by digitizing and curating the seminal report of Amerine and Winkler (1944), which provided grape harvest dates (GHDs), the quality of musts and wines, and wine tasting notes for 148 cultivars from 1935–1941 across five contrasting climatic regions of California. To put this dataset into a climate change context, we collected GHDs and must sugar content (°Brix) records from 1991 to 2018 for four representative cultivars in one of the five studied regions (Napa). Finally, we integrated meteorological data of the five regions during 1911–2018 and calculated bioclimatic indices important for grape. The resulting database is unique and valuable for assessing the fitness between cultivars across environments in order to mitigate the effects of climate change.

**Table:** 

Design Type(s)	Cultivars design • Regions design
Measurement Type(s)	Climate data • Harvest date • Quality • Tasting notes
Technology Type(s)	Phenology characterization • Quality determination
Sample Characteristic(s)	Grape harvest dates • °Brix • Tannin • Total acid • pH • Alcohol • Fixed acid • Extract
Measurement(s)	maximum air temperature • minimum air temperature • total soluble solids (^o^Brix) • must total acid • must pH • wine alcohol • wine extract • wine tannin • wine total acid • wine volatile acid
Technology Type(s)	weather station • a ^o^Brix hydrometer • titration with sodium hydroxide to a phenolphthalein end point • a quinhydrone electrode or a Beckman pH meter • hydrometer • a special 0° to 8° Balling hydrometer • the Association of Official Agricultural Chemists method • titration with phenolphthalein as an indicator • titration with pretreated wines by method II of the Association of Official Agricultural Chemists

## Background & Summary

Grape is one of the world’s most economically valuable fruit crops and grape quality is the foundation for high quality wines. Global warming has resulted in advanced maturity date, higher temperatures during ripening, higher sugar content, and lower acidity^1–4^, all contributing to changes in wine quality and style^5,6^. With continually increasing temperature expected in the near-future, growers can expect more far-reaching impacts on the sustainability of viticulture and typicality of wine in the coming decades^7,8^. Selecting diverse and well-adapted cultivars is critical to mitigate climate change effects, maintain (or even increase) sustainability, and ensure high-quality wines, because different cultivars have distinct sensitivities to temperature and require specific climate conditions (e.g. cool, warm, hot etc.) for producing premium quality grapes^9^. However, grape growing currently utilizes a surprisingly low amount of cultivar diversity. In fact, there are more than 3000 wine grape cultivars, but only 12 cultivars account for 70–90% of the total planting areas around the world^10^. The largely untapped cultivar diversity has a huge potential to help viticulture adapt to climate change.

Grapevine cultivars possess a vast genetic diversity in many essential traits, including phenology, which refers to the timing of the stages of plant development that occur during the vine’s annual growth cycle^11^. Main phenological stages for grapevine include budbreak, flowering, veraison (the onset of ripening), and maturity. Cultivars with distinct phenology will be differentially affected by the currently observed climate change driven shifts towards earlier development. For example, an early-maturing cultivar may suffer from heat stress during ripening and cause decrease in grape quality under warming climate condition, while those of late-ripening cultivars may have time to mature fully in areas where they were previously unable to ripen^12,13^. Moreover, earlier budbreak caused by warming climate may bring higher risks of spring frost for grapevines^14^. Therefore, phenology is vital in determining the suitability of a given cultivar to a particular climatic region. At present, there are very few comprehensive long-term phenological databases that include a diversity of environments and cultivars. These kinds of databases are needed to understand cultivar by environment interactions, describe the effects of global warming on the viticulture, and develop phenological models capable of predicting the plasticity of cultivar behaviour in the future. Note that France’s long tradition of recording phenology for wine grapes, researchers there have created an open-access database of observed grape harvest dates (GHDs) for a range of cultivars and sites across many decades^15,16^. These types of databases can inform strategies for grape-growers and wine-makers to mitigate the adverse impacts of a warming climate.

Phenology data should be integrated with information on grape composition and wine quality in order to accurately evaluate cultivar adaptability to specific environments. Previous researches have used climatic indices based mainly on temperature to establish past and future suitability, explore possible geographical shifts of vineyards, and investigate the relationships between growing season temperature, GHDs, and grape composition^17–20^. These studies help determine which cultivars are most suitable for a specific grape growing region and how cultivar suitability may change in the face of climate change.

California is home to some of the world’s top wine growing regions. The state on the west coast of the U.S. spans 1100 kilometers from north to south, providing a large diversity of climates for high-quality grape growing. The main producing areas consist of the Northern Coast, Central Coast, Central Valley, Sierra Nevada foothills and the Southern Coast. The North Coast of California is where many of the most famous wine regions are found, including Napa, Sonoma, and Mendocino. Temperatures are increasing in many of these regions although to date these increases appear to have benefited wine production^21,22^. With warmer temperatures in the future, suitable grape production areas could decline and/or be redistributed in California^23^. This will likely be a huge challenge for the grape industry as both established and new viticultural regions adapt to a changing environment.

In present study, we digitized and curated a unique dataset of GHDs, quality records of musts and wines, and wine tasting notes for 148 grape cultivars from 1935 to 1941 across five contrasting climatic regions^24^. These data were integrated with the corresponding climate data from 1911–2018 in order to determine cultivar suitability to specific climates. Finally, we complemented the dataset with both GHDs and must sugar content (°Brix) records under the past (1935–1941) and current (1991–2018) climates. This database can be used in the future to assess cultivar suitability, and evaluate climate change impact on GHDs and grape quality for the same set of cultivars across diverse climatic regions. The data can be combined with additional records to develop phenological and process-based growth models of grape by using GHDs and quality-related data.

## Method

### Site description

**Fig. 1:**
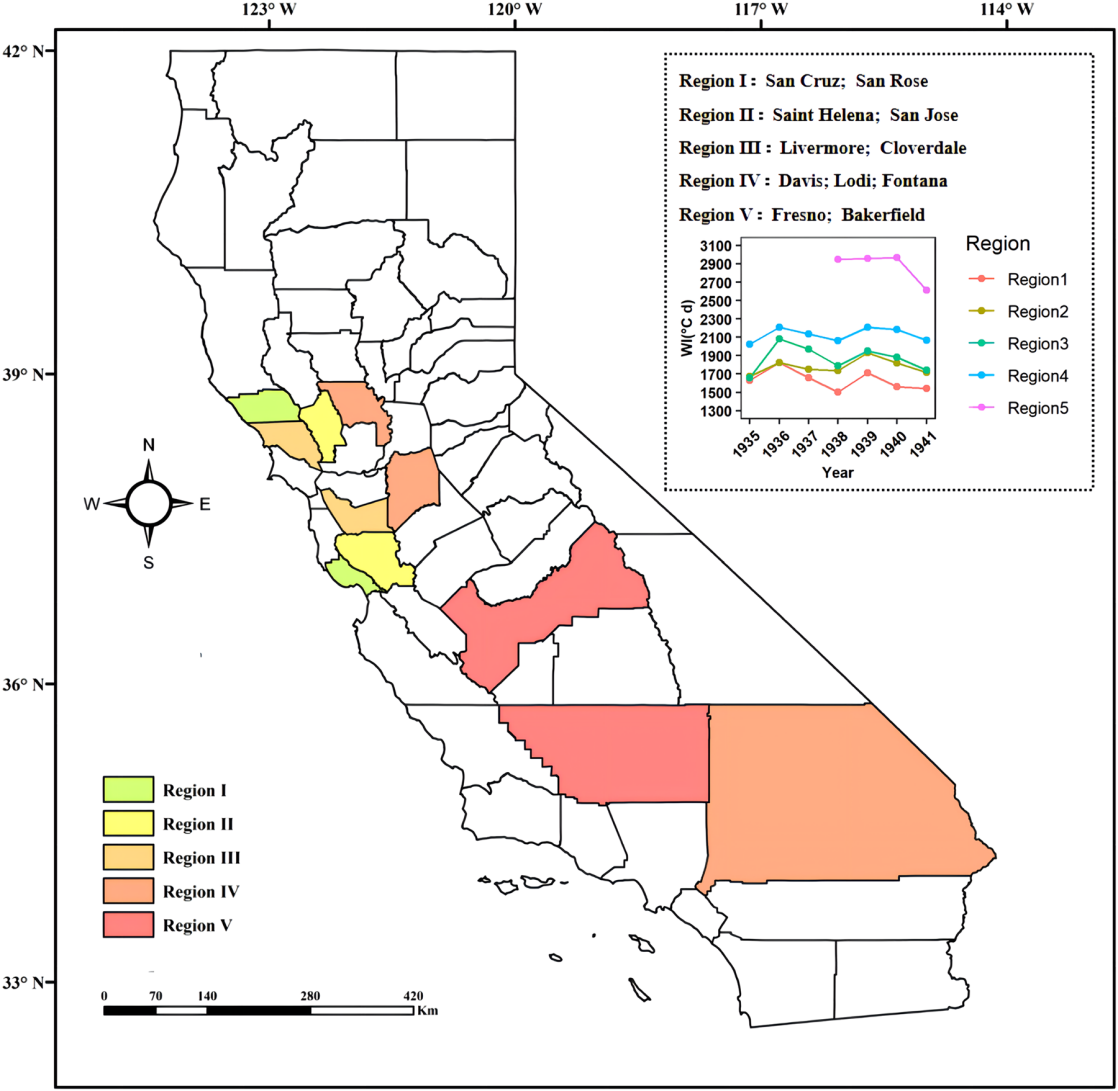
The locations of five climatic regions for wine grape classed by Winkler index in California. The insert plot represents the distinct Winkler index (WI) during 1935–1941 in five climatic regions.

The respective sites were classified into five climatic regions in California, containing San Cruz and San Rose in region 1, Saint Helena and San Jose in region 2, Livermore and Cloverdale in region 3, Davis, Lodi and Fontana in region 4, Fresno and Bakerfield in region 5 (Fig. 1). There were differences in annual mean temperature among five climatic regions, ranging from 14.3°C to 18.6°C. In each region, the GHDs, quality of musts and wines, and wine tasting notes were recorded for 148 cultivars from 1935 to 1941. Meanwhile, in region 2, namely in Napa, the GHDs and must sugar content (in °Brix) were recorded for four representative cultivars (Cabernet Sauvignon, Chardonnay, Merlot and Sauvignon Blanc) during 1991–2018.

### Climate data

The climate data was collected from five stations for over one hundred year-period (1911–2018), including daily average, maximum and minimum temperature (Table 1). Climate data was retrieved from the National Oceanic and Atmospheric Administration (NOAA)’s National Centers for Environmental Information (NCEI). The database from which the data was retrieved was the “Global Historical Climatology Network - Daily (GHCN-Daily), Version 3” (https://www1.ncdc.noaa.gov/pub/data/ghcn/daily/by_station/)^25,26^. Table 1 showed the search codes and names of five stations in the website. The climate data of region 1 and region 5 were for the periods of 1911–2011 and 1938–2018, respectively.

**Table 1:** Description of weather stations and time-span in five climatic regions.

Region	Station_name	Station_code	Latitude	Longitude	Altitude	Year
Region 1	SANTA_ROSA, CA	USC00047965	38.46 N	−122.71 E	51.0 m	1911–2011
Region 2	SAINT_HELENA, CA	USC00047643	38.51 N	−122.47 E	69.0 m	1911–2018
Region 3	LIVERMORE, CA	USC00044997	37.69 N	−121.81 E	120.0 m	1911–2018
Region 4	DAVIS_2_WSW_EXP_FARM, CA	USC00042294	38.53 N	−121.78 E	18.0 m	1911–2018
Region 5	BAKERSFIELD_AP, CA	USW00023155	35.43 N	−119.05 E	149.0 m	1938–2018

### Bioclimatic indices

Here, we presented seven temperature-related indices to explore the changing climate in five climatic regions during the last 100 years. We compared the changes of these indices between the past (1935–1941) and current climate conditions (1991–2018). Thereafter, four indices were chosen to describe annual changes, including average, maximum, minimum temperature and diurnal temperature range (DTR). Furthermore, other indices were used to analyse growing season temperature (GST), Winkler index (WI) and Huglin index (HI) for the grape-growing season^5,27,28^. The equations used to calculate the bioclimatic indices of grape-growing season are:

1 $$GST=\frac{{\sum }_{Apr1}^{Oct31}\frac{{T}_{max}+{T}_{min}}{2}}{n}$$

2 $$WI={\sum }_{Apr1}^{Oct31}\left(\frac{{T}_{max}+{T}_{min}}{2}-10\right)$$

3 $$HI={\sum }_{Apr1}^{Sep30}\left(\frac{{T}_{max}+{T}_{ave}}{2}-10\right)\times K$$

where *T*_*max*_, *T*_*min*_ and *T*_*ave*_ represent daily maximum, minimum and average temperatures, respectively. *K* is a length of day coefficient ranging from 1.02 to 1.06 between 40 and 50 of latitude in the northern hemisphere.

### Sample collection, harvest dates, quality of musts and wines measurement

Sample collection, harvest dates, quality of musts and wines measurement were detailed in the report of Amerine and Winkler^24^. Briefly, grape berries (22–220 kg) were picked in the morning from representative vines of variety collections or commercial vineyards by Amerine and Winkler^24^, as well as numerous vineyard owners. The harvest dates were recorded after picking. All grapes picked were crushed within 24 hours except for a few samples in 1935. The clear juice was taken after the coarse sediment had settled, in order to measure total soluble solids (°Brix), total acid (grams per 100 cc), and pH of must. The must was placed in an open oak fermenting tank. After fermentation, it was completed in a closed oak container. Then, the alcohol (percent by volume), extract (grams per 100 cc), tannin (grams per 100 cc), and fixed acid (grams per 100 cc) of wine were measured. The must °Brix was measured with a Brix hydrometer floating in a cylinder, must total acid was determined by titration with sodium hydroxide to a phenolphthalein end point, and must pH was measured with a quinhydrone electrode or a Beckman pH meter. In addition, wine alcohol was measured by the hydrometer and reported as percentage by volume, the extract and tannin of wine were measured by means of a special 0° to 8° Balling hydrometer and the Association of Official Agricultural Chemists method^24^. Note that the fixed acid of wine are equal to total acid minus volatile acid, where the total acid was measured by titration with phenolphthalein as an indicator while the volatile acid was determined also by titration with pretreated wines by method II of the Association of Official Agricultural Chemists^24^.

### Wine tasting notes

The purpose of wine tasting was to evaluate the cultivars based on the merits and defects of wine. The descriptive terms used for recording the results of the organoleptic examination contained appearance, color, odors, volatile acidity, total acidity, dryness, body, taste, smoothness and astringency, and general quality.

## Data Records

**Fig. 2:**
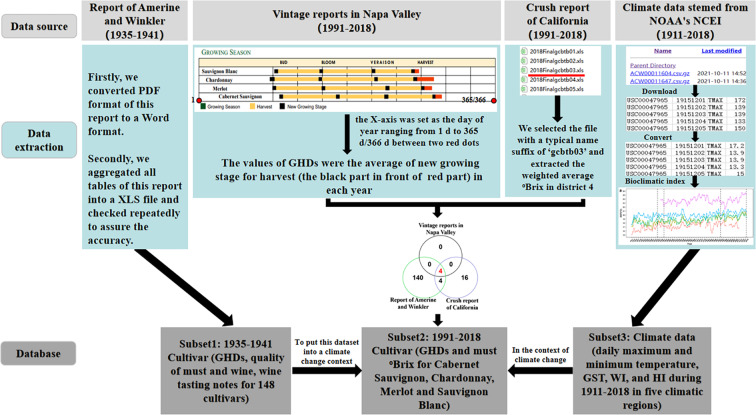
Flow chart of data integration. The venn diagram shows the cultivar complementarity among the report of Amerine and Winkler^24^, vintage report in Napa Valley^30^, and crush report of California^31^ and highlights that there are only four overlapping cultivars common to the three data sources.

This dataset was entered into three Excel spreadsheets and stored in the Figshare Digital Repository ^29^, including daily temperature conditions for different climatic regions, GHDs, quality of musts and wines, and wine tasting notes for different cultivars under diversity environment conditions (Fig. 2). They were digitized and curated from four resources, including climate data^25,26^, the seminal report of Amerine and Winkler^24^, Napa Valley vintage reports^30^, and the crush reports of California^31^.

**Fig. 3:**
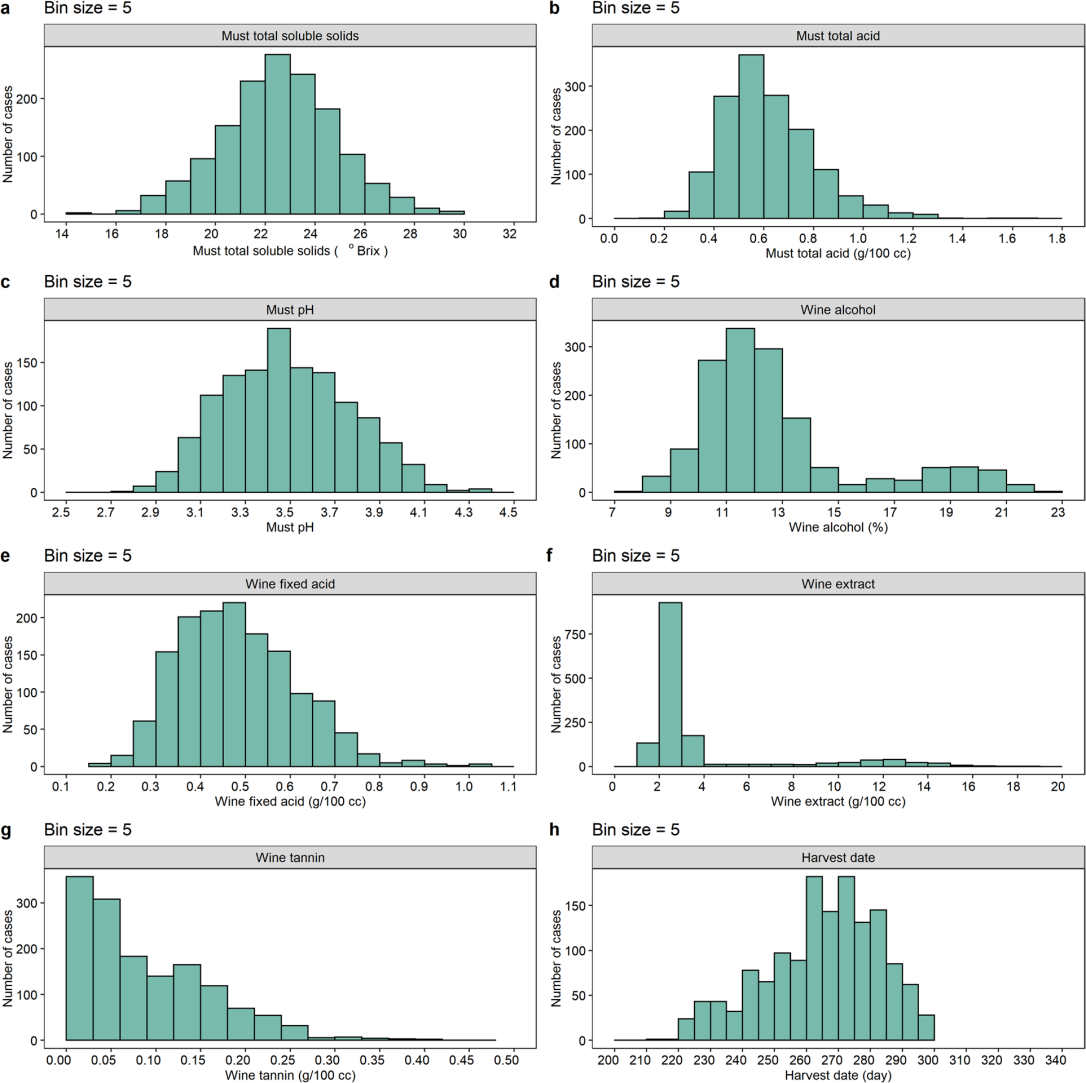
Distributions of must °Brix (**a**), must total acid (g/100 cc) (**b**), must pH (**c**), wine alcohol (%) (**d**), wine fixed acid (g/100 cc) (**e**), wine extract (g/100 cc) (**f**), wine tannin (g/100 cc) (**g**) and GHDs (day) (**h**) from 1935 to 1941 in five climatic regions of California.

The first and core subset was created by digitizing and curating the seminal report of Amerine and Winkler^24^, which provided grape harvest dates (GHDs), the quality of musts and wines, and wine tasting notes for 148 cultivars from 1935–1941 across five contrasting climatic regions of California (Fig. 3). This study not only explored the interrelations of environments and cultivars^24^ but also provided the foundation to establish the long-lasting and widely used viticultural zoning index, the Winkler index (WI)^27^. This index represents temperature characteristics over grapevine growing seasons for a given region and has been well recognized as one of the most important and reliable bioclimatic indices in viticulture. It has been cited at least 537 times when ‘Winkler index’ is searched in the Web of Science database. To explore whether the climate and grape performance of those regions studied in Amerine and Winkler^24^ have been altered over time, we collected GHDs and must °Brix from 1991 through 2018 for four overlapping cultivars (Cabernet Sauvignon, Chardonnay, Merlot and Sauvignon Blanc) in one of the five studied regions (Napa) to create the second and complementary subset of data. The choice of Napa and the four overlapping cultivars was based on data availability and complementarity with those reported in Amerine and Winkler^24^ (Fig. 2). After full exploration of available data about climate and grape traits in the previously studied regions and cultivars, we found only Napa had complete records of phenology and °Brix for four wine grapes (Cabernet Sauvignon, Chardonnay, Merlot and Sauvignon Blanc) during 1991–2021. These datasets were obtained from two sources. GHDs were obtained from the Napa Valley vintage reports^30^ and °Brix was derived from the crush reports of California in the website of United States Department of Agriculture (USDA)’s National Agricultural Statistics Service (NASS)^31^. In detail, there were four cultivars (Cabernet Sauvignon, Chardonnay, Merlot and Sauvignon Blanc) from 1991 to the present in the Napa Valley vintage reports, which described the key phenological periods of grapes, including budbreak, flowering, veraison, and GHDs. We digitized these vintage charts and extracted the GHDs by using the WebPlotDigitizer software^32^. When the GHDs were extracted, the X-axis was set as the day of year ranging from 1 d to 365 d/366 d (Fig. 2). The values of GHDs were the average of new growing stage for harvest in each year. In addition, the crush reports of California from 1976 to the present, including weighted average °Brix and weighted average dollars per ton, etc., were downloaded and unzipped. Secondly, we selected the third XLS file with a typical name suffix of ‘gcbtb03’, including °Brix of raisin grapes, table grapes, and wine grapes for white and red from district 1 to district 17 in California. We extracted the weighted average °Brix for wine grapes. Note that district 4 represented Napa, namely region 2 in Winkler’s zoning. We extracted GHDs and °Brix for four cultivars (Cabernet Sauvignon, Chardonnay, Merlot and Sauvignon Blanc) during the period of 1991–2018 in Napa, meanwhile, the period of 1991–2018 was divided into four periods (1991–1997, 1998–2004, 2005–2011, and 2012–2018) to compare with the past 7-year period (1935–1941).

The third subset is climate data in the Excel spreadsheet containing five sheets. Each sheet represents different climatic regions from region 1 to region 5. The source of raw data from which the data was retrieved was the “Global Historical Climatology Network - Daily (GHCN-Daily), Version 3” from the NOAA’s National Centers for Environmental Information (NCEI) (https://www1.ncdc.noaa.gov/pub/data/ghcn/daily/by_station/)^25,26^. The CSV files of climate data for each station can be searched based on the station_code in Table 1. There were 18 variables in the downloaded CSV file, while two temperature- related variables were used in this paper, including daily maximum and minimum temperature. The daily average temperature was then calculated as the arithmetic mean between daily maximum and minimum temperature. It is worth noting that the original data of daily maximum and minimum temperature are in tenths of degrees C, as indicated in the ‘GHCN-Daily README file’ (https://www1.ncdc.noaa.gov/pub/data/ghcn/daily/readme.txt)^33^. Furthermore, there were a few missing data for daily maximum and minimum temperature in the original data. The Python software was used to complement the data through calculating multi-year average value. Then, the complete data was applied to calculating three bioclimatic indices important for grape, including growing season temperature (GST), Winkler index (WI) and Huglin index (HI) for grape-growing seasons. The data of three bioclimatic indices calculated in each region were entered into corresponding sheets of Excel spreadsheet. All data can be found in the data set file stored in the Figshare Digital Repository^29^. The metadata for each data subset was shown in a specific sheet named ‘metadata’ in each subset xls file. Finally, we ensured that we had the right to release the final dataset under an open data license with the citation information indicated.

## Technical Validation

**Fig. 4:**
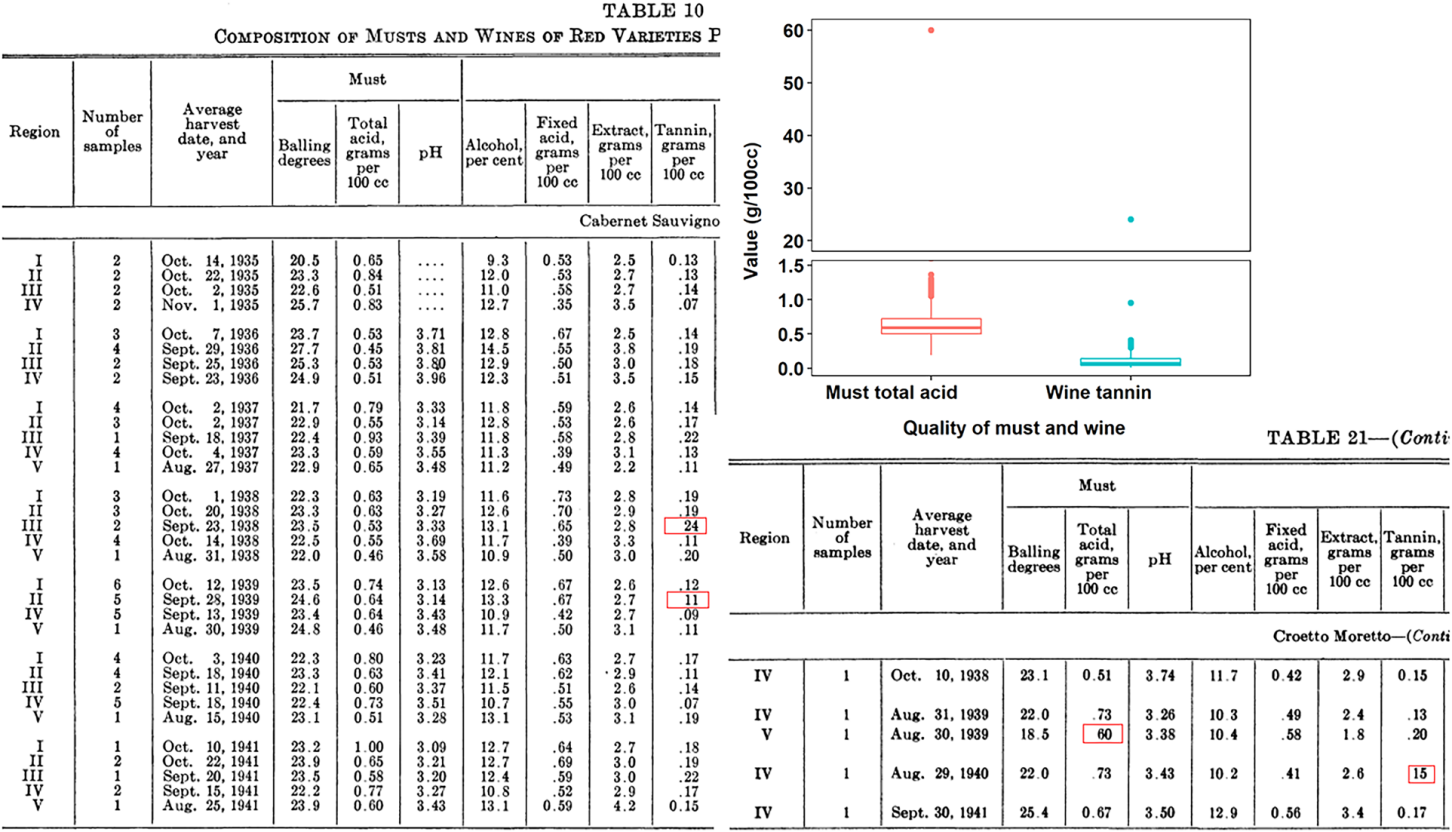
The suspicious data records (highlighted with red rectangle) in the report of Amerine and Winkler^24^.

We carefully checked the validity of climate data, GHDs, quality of musts and wines, and wine tasting notes recorded. We carried out manual validation checks to search suspicious data records before submitting data to the database. Shown in Fig. 4, we found that total acid and tannin of wine for Cabernet Sauvignon, Croetto Moretto, and Mission obviously deviated from most records being one or two magnitudes higher in comparison with other values in the original Table 10 and 21. And these discrepancies were most likely due to a miss typing of the decimal. Thus, we modified the tannin values of 24, 11, and 15 into 0.24, 0.11, and 0.15 with the unit of g/100 cc. Similarly, the total acid value of 60 in Table 21 (number in the original report) was changed into 0.60 with the unit of g/100 cc.

### Climatic indices

To quantify climatic variation and illustrate the warming temperature in five climatic regions, we calculated the change of annual average, maximum and minimum temperature, diurnal temperature range (DTR), growing season temperature (GST), Winkler index (WI) and Huglin index (HI) for the long-term series (Table 2). The annual average temperature, minimum temperature, GST, WI, and HI showed an increased trend during 1911–2018 in five climatic regions, while the change of annual maximum temperature was not significant and DTR decreased during 1911–2018 in five climatic regions. In conclusion, the changes of annual maximum, minimum temperature, and DTR suggested that temperature changes during the day and night were asymmetric.

**Table 2:** The range and average of annual average (T_ave_), maximum (T_max_), minimum (T_min_) temperature, diurnal temperature range (DTR), GST, WI and HI from 1911 to 2018 in five regions.

Factor	Region 1			Region 2			Region 3			Region 4			Region 5		
	Range	Average	∆	Range	Average	∆	Range	Average	∆	Range	Average	∆	Range	Average	∆
T_ave_	12.5–15.9	14.3	0.13	13.7–17.4	16.1	0.18	13.5–17.4	15.3	0.12	13.9–17.8	15.8	0.11	16.8–20.8	18.6	0.12
T_max_	19.3–24.3	22.0	−0.03	20.7–24.4	23.1	0.09	20.4–25	22.9	0.06	21.2–25.8	23.7	−0.0004	23.5–27.6	25.6	0.03
T_min_	4–9.6	6.6	0.31	5.2–9.9	8.7	0.26	5.4–10	7.7	0.19	4.6–9.8	7.8	0.22	9.9–14	11.7	0.2
DTR	12–18.2	15.4	−0.34	13–18.6	14.4	−0.16	12.4–18	15.3	−0.12	13.2–18.9	15.9	−0.23	11.8–16.7	13.9	−0.17
GST	15.1–18.8	17.2	0.14	16.4–20.8	18.6	0.19	17.2–20.9	18.9	0.16	17.6–21.7	20.1	0.12	21.5–25.6	23.7	0.11
WI	1099–1877	1546	30	1370–2307	1851	40	1552–2338	1912	34	1641–2499	2157	25	2472–3341	2937	24
HI	967–1684	1371	24	1171–1937	1575	38	1383–2064	1709	30	1504–2224	1957	20	2257–3005	2642	21

The ∆ represents the change trends of T_ave_, T_max_, T_min_, DTR, GST, WI and HI with the unit of °C per 10 year.

### Harvest dates, musts and wines analyses

**Fig. 5:**
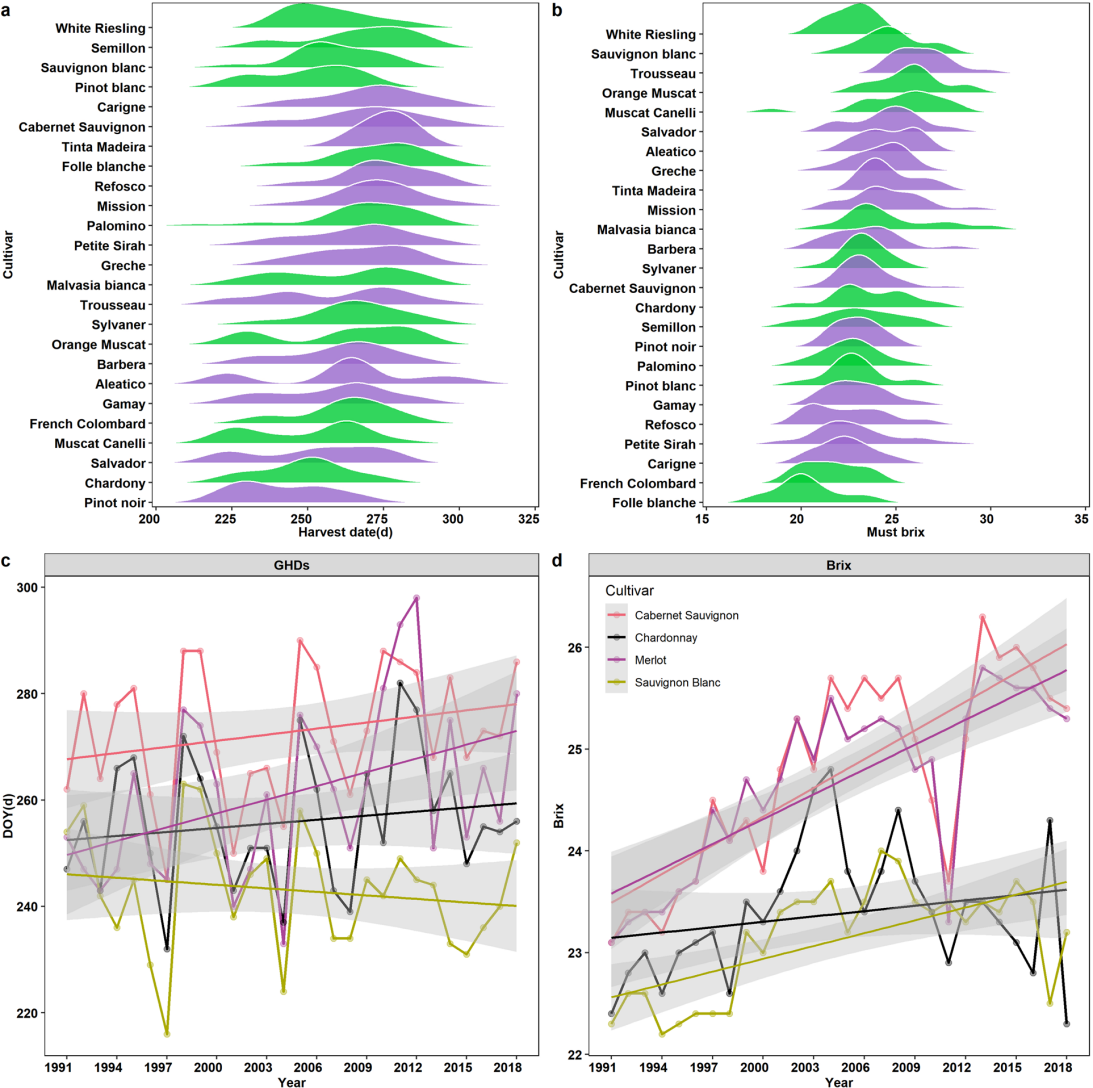
The ranges of GHDs (**a**) and must °Brix (**b**) from 1935 to 1941 for recommended cultivars in California. The purple and green represent red and white cultivars respectively in the density plot. The trends of GHDs (**c**) and °Brix (**d**) for Cabernet Sauvignon, Merlot, Chardonnay, and Sauvignon Blanc during 1991–2018 in California.

Here, we showed the whole data of GHDs, musts and wines analyses for recommended cultivars during 1935–1941 in five regions of California (Figs. 3 and 5). Most of the recorded variables followed a normal distribution in the first subset data (Fig. 3), with must °Brix levels ranging from 14.2° to 30.3° and GHDs ranging from 213 d to 336 d. We discovered the recommended cultivars possess the similar harvest window (Fig. 5a). In addition, we analyzed the changes of GHDs and °Brix for Cabernet Sauvignon, Chardonnay, Merlot and Sauvignon Blanc from 1991 to 2018 (Fig. 5c,d), meanwhile, the GHDs and °Brix of Cabernet Sauvignon and Sauvignon Blanc in region 2 were compared under the past and current climate conditions (Table 3). The results suggested that GHDs decreased first and then increased while °Brix showed an increased trend for a red cultivar (Cabernet Sauvignon). However, the change trends of GHDs and °Brix were similar for a white cultivar (Sauvignon Blanc), with both being lower in the current climate than that in the past climate.

**Table 3:** The GHDs and °Brix for Cabernet Sauvignon and Sauvignon Blanc in past (1935–1941) and current (1991–2018) climates of California.

Time period	Cabernet Sauvignon				Sauvignon Blanc			
	Range of GHDs (d)	Average value of GHDs (d)	Range of °Brix	Average value of °Brix	Range of GHDs (d)	Average value of GHDs (d)	Range of °Brix	Average value of °Brix
1935–1941	261~294	281	22.9~24.6	23.6	259~280	269	25.6~27.6	26.4
1991–1997	245~281	267	22.8~24.5	23.5	216~259	240	22.2~22.6	22.4
1998–2004	250~288	269	23.8~25.7	24.7	224~263	247	22.4~23.7	23.2
2005–2011	261~290	280	23.7~25.7	25.1	234~258	245	23.2~24.0	23.6
2012–2018	268~286	275	25.4~26.3	25.8	231~252	240	22.5~23.7	23.3

### Wine tasting notes

**Fig. 6:**
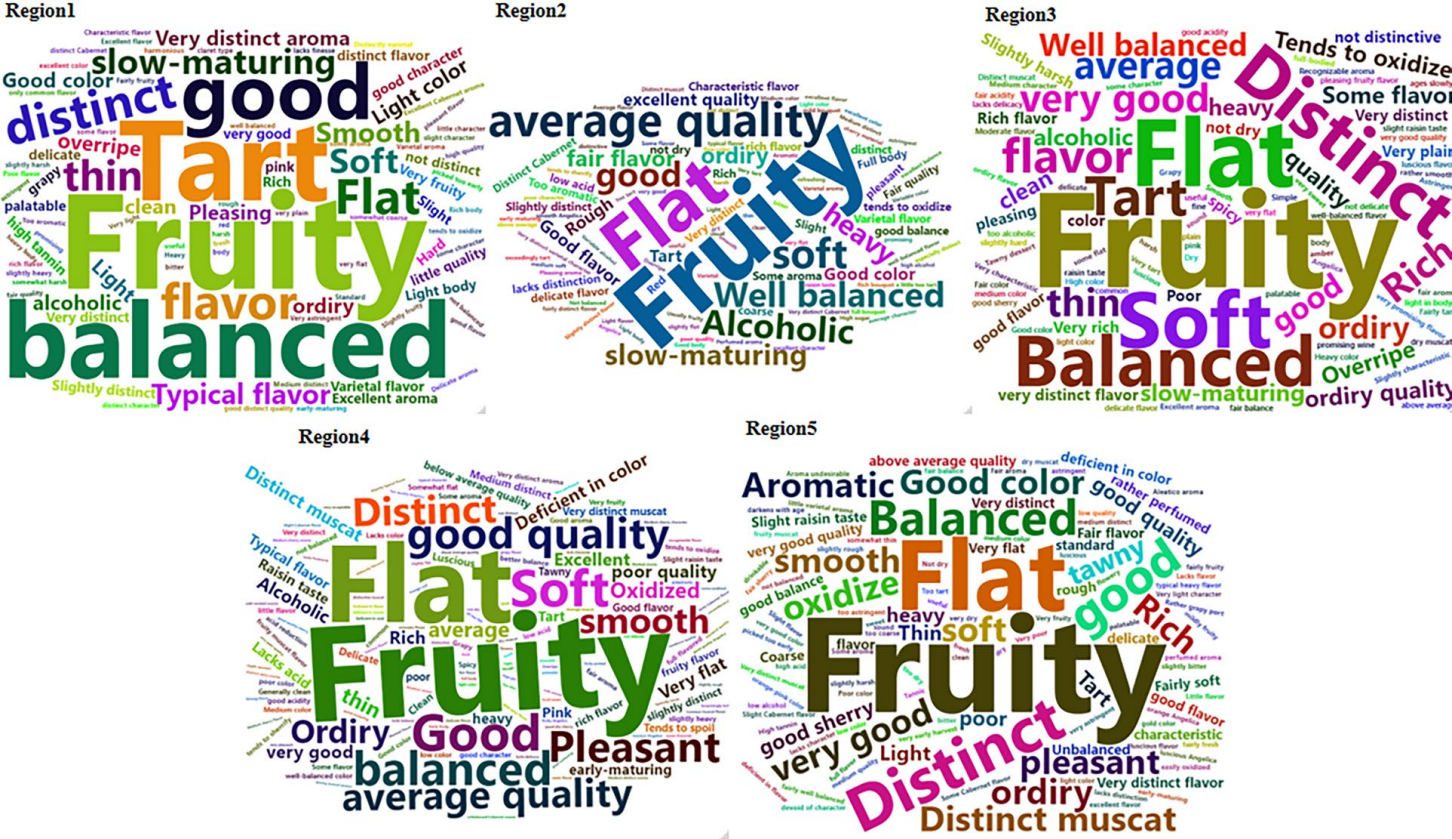
The word clouds of wine tasting notes for recommended cultivars in five climatic regions of California.

The text of wine tasting notes for recommended cultivars was explored. Firstly, we divided the original data of wine tasting notes into five regions based on the numbers of the sixth column in Subset1. Secondly, we sorted the original data of wine tasting notes for five regions into different words or phrases by the separator semicolon. Thirdly, the frequency of these words was counted in different regions. Finally, the R software was used to draw word clouds of wine tasting notes for the five regions^34^. These word clouds indicated that the characteristic of ‘fruity’ is the main feature of wines in California (Fig. 6). It is worth noting that wine tasting notes are less quantifiable than vintage/wine ratings, which have a numeric value for describing the overall quality of a vintage or wine. Jones *et al*.^5^ have analyzed a comprehensive set of vintage ratings as a function of climate change, and highlighted the warming temperature effects on wine qualities. However, vintage rating cannot tell why a vintage is excellent, good, or fair. Therefore, tasting notes analyzed with text mining provide novel insights into quality shifts through time and complement vintage/wine ratings.

## Data Availability

No custom code was used in this study.
